# High-Mobility Group Box-1 and Endothelial Cell Angiogenic Markers in the Vitreous from Patients with Proliferative Diabetic Retinopathy

**DOI:** 10.1155/2012/697489

**Published:** 2012-10-16

**Authors:** Ahmed M. Abu El-Asrar, Mohd Imtiaz Nawaz, Dustan Kangave, Marwan Abouammoh, Ghulam Mohammad

**Affiliations:** ^1^Department of Ophthalmology, College of Medicine, King Saud University, Riyadh 11411, Saudi Arabia; ^2^Department of Ophthalmology, King Abdulaziz University Hospital, Old Airport Road, P.O. Box 245, Riyadh 11411, Saudi Arabia

## Abstract

The aim of this study was to measure the levels of high-mobility group box-1 (HMGB1) in the vitreous fluid from patients with proliferative diabetic retinopathy (PDR) and to correlate its levels with clinical disease activity and the levels of vascular endothelial growth factor (VEGF), the angiogenic cytokine granulocyte-colony-stimulating factor (G-CSF), the endothelial cell angiogenic markers soluble vascular endothelial-cadherin (sVE-cadherin), and soluble endoglin (sEng). Vitreous samples from 36 PDR and 21 nondiabetic patients were studied by enzyme-linked immunosorbent assay. HMGB1, VEGF, sVE-cadherin, and sEng levels were significantly higher in PDR patients than in nondiabetics (*P* = 0.008; <0.001; <0.001; 0.003, resp.). G-CSF was detected in only 3 PDR samples. In the whole study group, there was significant positive correlation between the levels of HMGB1, and sVE-cadherin (*r* = 0.378, *P* = 0.007). In PDR patients, there was significant negative correlation between the levels of sVE-cadherin and sEng (*r* = −0.517, *P* = 0.0005). Exploratory regression analysis identified significant associations between active PDR and high levels of VEGF (odds ratio = 76.4; 95% confidence interval = 6.32–923) and high levels of sEng (odds ratio = 6.01; 95% confidence interval = 1.25–29.0). Our findings suggest that HMGB1, VEGF, sVE-cadherin and sEng regulate the angiogenesis in PDR.

## 1. Introduction

 Ischemia-induced angiogenesis and expansion of extracellular matrix in association with the outgrowth of fibrovascular membranes at the vitreoretinal interface is the pathological hallmark in proliferative diabetic retinopathy (PDR). Vascular endothelial growth factor (VEGF), an endothelial cell mitogen that also enhances vascular permeability, is thought to be the major angiogenesis factor in PDR [1]. In addition, strong evidence indicates that chronic low-grade inflammation is implicated in the pathogenesis of diabetic retinopathy [2, 3]. Sustained proinflammatory responses in diabetic retinopathy are often associated with angiogenesis [2–5]. The causal relationship between inflammation and angiogenesis is now widely accepted [6]. An emerging issue in diabetic retinopathy research is the focus on the mechanistic link between chronic, low-grade inflammation and angiogenesis.

 High-mobility group box-1 protein (HMGB1) was initially discovered as a nuclear chromatin-binding protein that stabilizes nucleosome formation and facilitates transcription. Necrotic cell death can result in passive leakage of HMGB1 from the cell as the protein is then no longer bound to DNA. In addition, HMGB1 can be actively secreted by different cell types, including activated monocytes and macrophages, mature dendritic cells, natural killer cells, and endothelial cells. Extracellular HMGB1 functions as a proinflammatory cytokine [6–10] and exhibits angiogenic effects [10–14]. HMGB1 signals through the receptor for advanced glycation end products (RAGE) leading to activation of the transcription factor nuclear factor kappa B (NF-*κ*B) and induces the expression of various leukocyte adhesion molecules and proinflammatory cytokines, chemokines, and angiogenic factors [6–9]. These findings suggest that HMGB1 might provide the mechanistic link between chronic low-grade inflammation and angiogenesis. In a previous report, we demonstrated that HMGB1 and RAGE were expressed by vascular endothelial cells and stromal cells in PDR fibrovascular epiretinal membranes and that there were significant correlations between the level of vascularization in PDR epiretinal membranes and the expression of HMGB1 and RAGE [15]. In addition, we demonstrated increased levels of HMGB1 in the vitreous samples from patients with PDR and that HMGB1 expression was upregulated in the retinas of diabetic mice. Moreover, there were significant correlations between the vitreous levels of HMGB1 and the levels of the inflammatory biomarkers monocyte chemoattractant protein-1 (MPC-1) and soluble intercellular adhesion molecule-1 (sICAM-1) [16].

 Over the years, great effort has been made to find specific markers for the angiogenic endothelial cells that can be exploited by vascular targeting agents. Among these markers, the endothelial cell activation markers vascular-endothelial-(VE-) cadherin and endoglin (Eng) stand out as reliable biomarkers of angiogenesis activity. VE-cadherin is a cell adhesion molecule localized at the endothelial junction. VE-cadherin plays a key role in angiogenesis, signaling, endothelial cell survival, and endothelial cell barrier function. The regulation of its biological activity may be the central mechanism in normal or pathological angiogenesis [17, 18]. This molecule can be shed from the cell surface and elevated serum levels of soluble VE-chaderin (sVE-cadherin) seem to be a reliable marker of endothelial angiogenic activity and/or injury [19–25]. 

 Endoglin (Eng) (also known as CD105), a type I transmembrane glycoprotein highly expressed on proliferating vascular endothelial cells, has been identified as an accessory receptor for transforming growth factor-*β* (TGF-*β*) and is essential for angiogenesis. Eng is expressed at low to nondetectable levels in resting endothelial cells within normal tissues, but its expression strongly increases in vascular endothelial cells in sites of active angiogenesis during embryogenesis, in inflamed tissues, in healing wounds, and in tumor vessels. Therefore, Eng detection is used as a marker to analyze angiogenesis and microvascular density in tumors and has been found to be an independent prognostic indicator. Expression of Eng can be induced by hypoxia and is also upregulated in ischemic tissues [26, 27]. Furthermore, a soluble form of Eng (sEng) has been observed in the serum of patients with different types of solid malignancies [28] and of pregnant women suffering from preeclampsia [29]. Circulating levels of sEng were found to be a reliable biomarker that correlates with disease severity and has prognostic significance [28, 29]. This soluble form, which results from partial shedding of the membrane-bound form of Eng by the matrix metalloproteinase-14 (MT1-MMP) [30], has been proposed to act as a scavenger or trap for circulating TGF-*β* family ligands such as bone morphogenetic proteins 9 and 10, thus impairing binding to their physiological receptors indicating an important role of sEng in the regulation of angiogenesis [31].

 The aim of this study was to measure the levels of HMGB1 in the vitreous fluid from patients with PDR and to correlate its levels with clinical disease activity and vitreous levels of VEGF, the angiogenic cytokine granulocyte-colony stimulating factor (G-CSF) [32–34] and the endothelial cell angiogenic markers sVE-cadherin and sEng.

## 2. Materials and Methods

### 2.1. Vitreous Samples

Undiluted vitreous fluid samples (0.3–0.6 mL) were obtained from 36 patients with PDR and 21 patients with rhegmatogenous retinal detachment (RD) without proliferative vitreoretinopathy during pars plana vitrectomy. The indications for vitrectomy in patients with PDR were traction retinal detachment and/or nonclearing vitreous hemorrhage. In patients with PDR, the severity of retinal neovascular activity was graded clinically at the time of vitrectomy using previously published criteria [35]. Neovascularization was considered active if there were visible perfused new vessels on the retina or optic disc present within tractional epiretinal membranes. Neovascularization was considered inactive (involuted) if only nonvascularized, white fibrotic epiretinal membranes were present. Active PDR was present in 19 patients and inactive PDR was present in 17 patients. Vitreous samples were collected undiluted by manual suction into a syringe through the aspiration line of vitrectomy, before opening the infusion line. The samples were centrifuged (500 rpm for 10 min, 4°C) and the supernatants were aliquoted and frozen at −80°C until assay. The study was conducted according to the tenets of the Declaration of Helsinki, and informed consent was obtained from all patients. The study was approved by the Research Centre, College of Medicine, King Saud University.

### 2.2. Enzyme-Linked Immunosorbent Assay Kits

 Enzyme-linked immunosorbent assay (ELISA) kits for human VE-cadherin (Human VE-cadherin, Cat No: DCADV0), human VEGF (Human vascular endothelial growth factor, Cat No: SVE00), human Eng (Human Endoglin/CD105, Cat No. DNDG00) and human G-CSF (Human granulocyte-colony stimulating factor, Cat No: DCS50), were purchased from R&D Systems, Minneapolis, MN, USA. The ELISA kit for HMGB1 (human high-mobility group box-1, Cat No: ST51011) was purchased from IBL International GMBH, Hamburg, Germany. 

The minimum detection limit of each ELISA kit for VE-Cadherin, VEGF, Eng, G-CSF, and HMGB1 is 113, 9, 7, 20, and 100 picograms/mL (pg/mL), respectively. The ELISA plate readings were done using FLUOstar Omega-Miroplate reader from BMG Labtech, Offenburg, Germany. 

### 2.3. Measurement of VE-Cadherin, VEGF, Eng, G-CSF, and HMGB1

The quantification of human VE-cadherin, VEGF, Eng, G-CSF, and HMGB1 in  the vitreous fluid was determined using ELISA kits according to the manufacturer's instruction. For each ELISA kit, the undiluted standard served as the highest concentration and calibrator diluents served as the blank. Depending upon the detection range for each ELISA kit, vitreous samples were either directly used or diluted with calibrator diluents supplied with ELISA kit. 

For the measurement of VE-caherin and VEGF, 100 *μ*L of 5-fold and 2-fold diluted vitreous (sample diluents, supplied with the kit) was used in the respective ELISA assay for their analysis. For measurement of Eng and G-CSF, 100 *μ*L of undiluted vitreous was used and added to the wells of respective ELISA plates. For the quantification of HMGB1 within the high sensitivity range, 50 *μ*L of diluents buffer (Dilbuf, IBL International) was added to each well of microtiter plate followed by the addition of 50 *μ*L of standard, positive control, and vitreous fluid. 

As instructed in the kit manual, samples were incubated into the each well of ELISA plates. The antibody against VE-cadherin, VEGF, Eng, G-CSF, and HMGB1,conjugated to horseradish peroxidase was added to each well of the ELISA plate. After incubation, substrate mix solution was added for colour development. The reaction was stopped by the addition of 2N sulfuric acid and optical density was read at 450 nm in microplate reader. Each assay was performed in duplicate. Using the 4-parameter fit logistic (4-PL) curve equation, the actual concentration for each sample was calculated. For the vitreous fluid that has been diluted, the concentration for each sample was calculated after multiplying with the dilution factors to get the actual reading for each sample.

### 2.4. Statistical Methods

Because of the large variances that we had in our data, we used the non-parametric Mann-Whitney test to compare means from two independent groups, and the nonparametric Kruskal-Wallis test was used for conducting Analysis of Variance (ANOVA) to compare means from more than two independent groups. Correlation between continuous variables was investigated by computation of the Pearson correlation coefficient. A *P* value less than 0.05 indicated statistical significance. Post-ANOVA pairwise comparisons of means were conducted using the Kruskal-Wallis test. For three groups, the critical *Z*-value for determining statistical significance was *Z* = 2.39. Exploratory logistic regression analysis involving forcing entry, into a logistic model, the variables of interest, was conducted to discover whether active PDR was associated with high or low levels for the variables that were investigated. The mean level of each variable was used as the cut-off value for high versus low levels. SPSS version 15 and programs LR and 3S from Bio-Medical Data Processing Version 2007 (BMDP 2007) Statistical Software (Cork Technology Pack, Model Farm Road, Cord, Ireland) were used for the statistical analyses.

## 3. Results

### 3.1. Levels of Angiogenesis Biomarkers in Vitreous Samples

 HMGB1, sVE-cadherin, and sEng were detected in all vitreous samples from patients with PDR and nondiabetic patients. VEGF was detected in 36 (90%) vitreous samples from patients with PDR and in 10 (45%) vitreous samples from nondiabetic patients. G-CSF was detected in only 3 (7.5%) vitreous samples from patients with PDR and in 6 (27%) vitreous samples from nondiabetic patients.

 The mean levels of HMGB1, VEGF, sVE-cadherin, and sEng in vitreous samples from PDR patients were significantly higher than those in nondiabetic patients (*P* = 0.008; *P* < 0.001; *P* < 0.001; *P* = 0.003, resp.; Mann-Whitney test) (Table 1).

### 3.2. Relationship between Angiogenesis Biomarkers and Activity of PDR

 Comparison of mean levels of angiogenesis biomarkers among active PDR patients, inactive PDR patients, and nondiabetic patients was conducted using the Kruskal-Wallis test and the results are shown in Table 2. Mean levels differed significantly between the 3 groups from HMGB1 (*P* = 0.028), VEGF (*P* < 0.001), sVE-cadherin (*P* < 0.001), and sEng (*P* = 0.006). Post-ANOVA pairwise comparisons of means indicated that mean HMGB1 level was significantly higher in patients with active PDR than in nondiabetic patients (*Z* = 2.53). For VEGF, the mean levels were significantly higher in patients with active PDR than that in inactive PDR patients and nondiabetic patients (*Z* = 3.88; *Z* = 5.46, resp.). For sVE-cadherin, the mean levels were significantly higher in patients with active PDR and patients with inactive PDR than those in nondiabetic patients (*Z* = 4.72; *Z* = 4.42, resp.). For sEng, the mean level in patients with inactive PDR was significantly higher than that in nondiabetic patients (*Z* = 3.16).

### 3.3. Correlations

 In the whole study group, there was a significant positive correlation between vitreous fluid levels of HMGB1 and sVE-cadherin (*r* = 0.378, *P* = 0.007). In PDR patients, there was a significant negative correlation between vitreous fluid levels of sVE-cadherin and sEng (*r* = −0.517, *P* = 0.005). 

### 3.4. Logistic Regression Analysis

 We conducted exploratory logistic regression analysis to investigate further the association between the angiogenesis biomarkers and active PDR. Active PDR was significantly associated with high levels of VEGF (odds ratio = 76.4; 95% confidence interval = 6.322–923) and high levels of sEng (odds ratio = 6.01; 95%  confidence interval = 1.25–29.0).

## 4. Discussion

 In the present study, the levels of HMGB1, VEGF, sVE-cadherin, and sEng were significantly higher in the vitreous fluid from PDR patients compared with nondiabetic patients. In contrast, G-CSF was detected in only few samples consistent with a previous study [36]. There was a significant positive correlation between the vitreous levels of HMGB1 and sVE-cadherin in the whole patient group and a significant negative correlation between sVE-cadherin and sEng in patients with PDR. Among the angiogenic factors that we investigated, VEGF and sEng had a stronger influence on the activity of PDR than the other factors.

 In the present study, HMGB1 levels were significantly elevated in the vitreous fluid from patients with PDR. Furthermore, the levels were higher in patients with active PDR compared with patients with quiescent PDR. In a previous study, we demonstrated that HMGB1 expression was upregulated in the retinas of diabetic mice [16]. Similarly, increased vascular [37] and renal [38] HMGB1 expression was recently demonstrated in diabetic animals. In addition, hyperglycemia-induced reactive oxygen species production increased the expression of HMGB1 and RAGE in endothelial cells [39]. In patients with type 1 diabetes, serum HMGB1 levels were positively associated with markers of low-grade inflammation and endothelial dysfunction. In addition, higher serum HMGB1 levels were associated with greater prevalence and severity of albuminuria [40]. Activation of HMGB1/RAGE signaling axis is important in promoting proinflammatory pathways considered to play an important role in diabetes-induced retinal vascular inflammation. In endothelial cells, HMGB1 induces the expression of RAGE and adhesion molecules, such as ICAM-1, vascular cells adhesion molecule-1, and E-selectin, to release tumor necrosis factor-*α* (TNF-*α*), G-CSF, interleukin-8, and MCP-1 and to increase neutrophil adhesion. This proinflammatory phenotype was mediated by the activation of NF-*κ*B and was RAGE dependent as it was inhibited by antibodies directed toward RAGE [7–10]. In our laboratory, we recently demonstrated that intravitreal administration of HMGB1 to normal rats induced significant upregulation of ICAM-1, HMGB1, and RAGE and NF-*κ*B activation in the retina (Mohammad et al., unpublished data). In turn endothelial cells secrete HMGB1 in response to TNF-*α* treatment [41], suggesting a role for HMGB1 in positive feedback loop promoting inflammation. Recently, HMGB1 has been recognized as an angiogenic cytokine [10–14]. HMGB1 treatment of endothelial cells induced a proangiogenic gene expression program evidenced by the induction of VEGF and its receptors, platelet-derived growth factor receptors, integrins and matrix metalloproteinases [10]. In addition, HMGB1 induced endothelial cell migration, and sprouting [10]. HMGB1 was also identified as a specific marker of tumor endothelium [14] and as a tumor angiogenesis marker [10]. Moreover, anti-HMGB1 antibodies inhibited tumor angiogenesis [10]. Another interesting role of HMGB1 in neovascularization is its ability to attract endothelial progenitor cells to sites of tissue injury and tumors to improve neovascularization in a RAGE-dependent manner [13].

 Several studies demonstrated that sVE-cadherin serum levels may reflect the intensity of angiogenesis. sVE-cadherin serum level was increased in untreated multiple myeloma patients and decreased after chemotherapy in patients in remission [19]. Similarly, circulating sVE-cadherin levels were increased in pregnant women (a physiological condition associated with increased angiogenesis) and cancer patients and were particularly increased in patients affected by hematological malignancies and decreased to normal values in patients achieving complete remission [20]. Reverse transcriptase-polymerase chain reaction was used to profile gene expression of proteins closely associated with angiogenesis. Results showed 10-fold increase in VE-cadherin during angiogenesis [25]. These findings are in agreement with another study that demonstrated that VE-cadherin was a selective marker for assessing microvessel density in breast cancer [42]. Serum sVE-cadherin levels were also increased in other pathologic states associated with endothelial dysfunction such as Behçet's disease [23], rheumatoid arthritis [21], coronary atherosclerosis [22], and ovarian hyperstimulation syndrome [24]. *In vitro* studies demonstrated that treatment of endothelial cells with TNF-*α* [21], VEGF [43], matrix metalloproteinase-9 [44], and the diabetic metabolite advanced glycation end products [44] resulted in shedding of the VE-cadherin extracellular domain and loss of cell-cell contact which may lead to increased vascular permeability. The present study is the first report documenting increased levels of sVE-cadherin in the vitreous fluid from patients with PDR. In addition, our analysis showed a significant positive correlation between the vitreous levels of HMGB1 and sVE-cadherin. It is well established that endothelial dysfunction is a key feature of diabetic retinopathy [44]. On the basis of our findings, we propose that elevated levels of sVE-cadherin in the vitreous fluid from patients with PDR could be a reflection of endothelial cell activation or injury associated with angiogenesis, inflammation, and breakdown of the inner blood-retinal barrier.

 The current study is the first to demonstrate that sEng is significantly upregulated in the vitreous fluid from patients with PDR. Our results are consistent with a previous report showing that plasma sEng concentration could serve as an indicator of diabetes-associated vascular pathologies such as retinopathy, hypertension, endothelial dysfunction, and cardiovascular risk [45]. Similarly, another study demonstrated that sEng could be a marker to predict cardiovascular events in patients with chronic coronary artery disease [46]. In addition, Li et al. [28] showed that plasma sEng is a valuable surrogate angiogenic marker for identifying breast cancer patients who are at high risk of developing metastasis. In a previous study, we demonstrated that Eng was expressed by vascular endothelial cells in PDR fibrovascular epiretinal membranes [47]. Therefore, it is possible that the increase in sEng in the vitreous fluid from patients with PDR resulted from Eng proteolytic shedding of the membrane-bound form associated with angiogenesis. A previous study showed elevated levels of matrix metalloproteinase-14 in the retinas of diabetic animals [48]. Matrix metalloproteinase-14 was shown in a previous report to induce shedding of the membrane-bound form of Eng [30]. Among the studied biomarkers of angiogenesis, exploratory logistic regression analysis revealed that higher levels of VEGF and sEng were associated with active PDR. These findings suggest that sEng may also represent a surrogate marker of angiogenic activity in PDR.

 Endothelial dysfunction is a major characteristic of patients with diabetic retinopathy [44]. Several studies demonstrated that sEng plays an important role in endothelial cell function and in regulating angiogenesis. Forced expression of sEng increased vascular permeability. *In vitro* studies on endothelial cell lines showed that sEng interferes with TGF-*β* signaling and endothelial nitric oxide activation and thereby causes endothelial dysfunction. sEng also seems to be a regulator of vascular tone, as administration of sEng to mice induces an increase in arterial pressure by increasing vascular resistance [49]. Recently, Walshe et al. [50] demonstrated that sEng increased vascular and neural cell apoptosis in the retina, which was associated with decreased retinal function and breakdown of the blood-retinal barrier. In addition, *in vitro* and *in vivo* studies demonstrated that sEng is capable of inhibiting angiogenesis [30, 31, 49]. Our analysis demonstrated a significant negative correlation between sEng levels and the levels of sVE-cadherin in the vitreous from patients with PDR. These findings suggest a lower angiogenic activity in patients with higher levels of sEng and that the upregulation of sEng in the vitreous fluid from patients with PDR may be a protective antiangiogenesis eye response to suppress progression of PDR. 

 In conclusion, these data suggest that, along with HMGB1 and VEGF, sVE-cadherin and sEng might play a role in the pathophysiology of PDR. In addition, sVE-cadherin and sEng might be valuable angiogenic markers for PDR. 

## Figures and Tables

**Table 1:** Comparisons of mean angiogenesis biomarker levels in proliferative diabetic retinopathy (PDR) and rhegmatogenous retinal detachment (RD) patients.

Disease group	HMGB1 (ng/mL)	VEGF (ng/mL)	sVE-cadherin (ng/mL)	sEng (ng/mL)
PDR (*n* = 36)	5.69 ± 8.5	0.85 ± 1.2	77.3 ± 63.5	3.64 ± 1.8
RD (*n* = 14)	1.70 ± 2.10	0.04 ± 0.1	10.7 ± 9.6	2.22 ± 0.7
*P* value (Mann-Whitney test)	0.008*	<0.001*	<0.001*	<0.003*

*Statistically significant at 5% level of significance.

HMGB1: high-mobility group box-1; VEGF: vascular endothelial growth factor; sVE-cadherin: soluble vascular endothelial-cadherin; sEng: soluble endoglin.

**Table 2:** Comparisons of mean angiogenesis biomarker levels in proliferative diabetic retinopathy (PDR) patients with or without active neovascularization.

Disease group	HMGB1 (ng/mL)	VEGF (ng/mL)	sVE-cadherin (ng/mL)	sEng (ng/mL)
Active PDR (*n* = 19)	7.28 ± 11.1	1.67 ± 1.4	75.8 ± 53.4	3.28 ± 1.9
Inactive PDR (*n* = 17)	4.02 ± 4.1	0.18 ± 0.4	78.8 ± 74.9	4.04 ± 1.6
RD (*n* = 21)	1.70 ± 2.1	0.04 ± 0.1	10.7 ± 9.6	2.22 ± 0.7
*P* value (ANOVA)	0.028*	<0.001*	<0.001*	0.006*

*Statistically significant at 5% level of significance.

HMGB1: high-mobility group box-1; VEGF: vascular endothelial growth factor; sVE-cadherin: soluble vascular endothelial-cadherin; sEng: soluble endoglin; RD: rhegmatogenous retinal detachment.
